# *JLigand*: a graphical tool for the *CCP*4 template-restraint library

**DOI:** 10.1107/S090744491200251X

**Published:** 2012-03-17

**Authors:** Andrey A. Lebedev, Paul Young, Michail N. Isupov, Olga V. Moroz, Alexey A. Vagin, Garib N. Murshudov

**Affiliations:** a CCP4, STFC Rutherford Appleton Laboratory, Harwell Oxford, Didcot OX11 0QX, England; b York Digital Library, University of York, Heslington, York YO10 5DD, England; c Henry Wellcome Building for Biocatalysis, Biosciences, College of Life and Environmental Sciences, University of Exeter, Stocker Road, Exeter EX4 4QD, England; d Structural Biology Laboratory, University of York, Heslington, York YO10 5DD, England; e Structural Studies Division, MRC Laboratory of Molecular Biology, Hills Road, Cambridge CB2 0QH, England

**Keywords:** macromolecular refinement, restraint library, molecular graphics

## Abstract

Biological macromolecules are polymers and therefore the restraints for macromolecular refinement can be subdivided into two sets: restraints that are applied to atoms that all belong to the same monomer and restraints that are associated with the covalent bonds between monomers. The *CCP*4 template-restraint library contains three types of data entries defining template restraints: descriptions of monomers and their modifications, both used for intramonomer restraints, and descriptions of links for intermonomer restraints. The library provides generic descriptions of modifications and links for protein, DNA and RNA chains, and for some post-translational modifications including glycosylation. Structure-specific template restraints can be defined in a user’s additional restraint library. Here, *JLigand*, a new *CCP*4 graphical interface to *LibCheck* and *REFMAC* that has been developed to manage the user’s library and generate new monomer entries is described, as well as new entries for links and associated modifications.

## Abstract 2

The *CCP*4 template-restraint library defines restraints for biopolymers, their modifications and ligands that are used in macromolecular structure refinement. *JLigand* is a graphical editor for generating descriptions of new ligands and covalent linkages.

## 1. *CCP*4 library of template restraints

### 1.1. Overview

In the early days of the CCP4 project (Collaborative Computational Project, Number 4, 1994 ▶; Winn *et al.*, 2011 ▶), the least-squares minimization program *PROLSQ* (Hendrickson & Konnert, 1980 ▶) was adopted as the main macromolecular refinement tool within the project. *PROLSQ* required a stand-alone program *PROTIN* which analyzed the atomic coordinate data and identified the atoms and ideal geometric values involved in the individual stereochemical restraints for the refinement. The ideal geometric values for amino acids and some ligand compounds were stored in the library file protin.dic. Every time a user needed to create a description of a novel ligand he had to manually prepare a list of its ideal values in *PROTIN* dictionary format with the help of the program *MAKEDICT* (Collaborative Computational Project, Number 4, 1994 ▶) and append it to the dictionary. The *PROTIN* dictionary was extensively modified at the beginning of the 1990s to conform to the Engh and Huber parameters (Engh & Huber, 1991 ▶). Earlier versions of the maximum-likelihood refinement program *REFMAC* (Murshudov *et al.*, 1997 ▶) also used *PROTIN* for preparation of stereochemical restraints. However, the limitations of the *PROTIN* dictionary incited the *REFMAC* developers to give up using *PROTIN* and to design a more flexible library of template restraints and incorporate restraints preparation into *REFMAC*.

The *CCP*4 library of template restraints (further referred to as the restraint library, but also known as the monomer library; Vagin *et al.*, 2004 ▶) originates from the library which was developed for the Protein Data Bank (PDB; Bernstein *et al.*, 1977 ▶) in order to store descriptions of ligands found in macromolecular structures. Since then, the restraint library and its PDB analogue the Chemical Components Dictionary (Dimitropoulos *et al.*, 2006 ▶) have diverged, although both use the same CIF format and have some common or similar entry types. The Chemical Components Dictionary is updated concurrently with the release of each coordinate entry containing a new ligand, while the *CCP*4 restraint library is updated less frequently: only with the main releases of the *CCP*4 suite. Additionally, the restraint library contains all of the information required for macromolecular structure refinement with *REFMAC* (Murshudov *et al.*, 2011 ▶). Along with descriptions of atoms and bond orders, it stores information about covalent bond lengths and angles, torsion angles and lists of chiral centres and planar groups. Another important feature of the restraint library is that it also provides definitions of restraints for linkages between the monomers in polymers. While the restraint library was originally developed for *REFMAC*, the principles of organization and format of the library were subsquently adapted for use in *PHENIX* (Moriarty *et al.*, 2009 ▶) and *BUSTER* (Bricogne *et al.*, 2011 ▶). The library is also used in *Coot* (Emsley *et al.*, 2010 ▶) for input of data required for real-space refinement of ligands and in *CCP*4*mg* (Potterton *et al.*, 2004 ▶).

The restraint library contains three main types of entries: monomers, modifications and links. Monomer entries define the molecular graph, atom names and template restraints for isolated compounds, including restraints for bonds, angles, torsion angles, planes and definitions of chiral centres. Modification and link entries are required to generate restraints for modified monomers and polymers and are discussed in more detail in the next section. There are also several auxiliary entries, including one defining atom energy types used by the program *LibCheck* (Vagin *et al.*, 1998 ▶, 2004 ▶) to generate new descriptions of monomers.

Currently, the program *Sketcher*, which is part of the *CCP*4*i* interface (Potterton *et al.*, 2003 ▶), is used as a graphical interface for *LibCheck*. While *Sketcher* is well adapted for generation of new monomer entries from scratch or from existing monomer entries, it is not possible to use it for the creation of link descriptions. Therefore, *JLigand*, a more versatile graphical interface for *LibCheck*, has been developed.

### 1.2. Covalent modifications and linkages

Template restraints for covalent linkages between monomers are defined in the restraint library using modification and link library entries. This mechanism was developed to describe changes resulting from chemical reactions, including polymerization reactions. In particular, modifications describe changes in monomers, while links allow joining of the modified monomers together. For example, a generic modification ‘DEL-OXT’ describes the deletion of carboxyl atom OXT from an amino acid and updates geometrical restraints around the deleted atom. This modification can be represented by the following scheme.
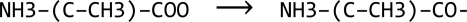


Similarly, the generic peptide modification ‘NH1’ describes the deletion of an H atom and a proton from a protonated amino group.
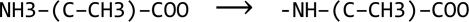


At this point, the generic link ‘TRANS’ can be applied to two modified peptides to generate template restraints for the covalent linkage between them.
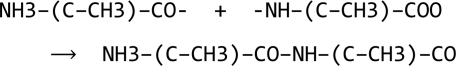


The mechanism described above may look as if it has an unnecessary complexity. It can be demonstrated why it nevertheless makes sense. Let us consider two approaches. In the first, only one type of data is available: the monomers. Let us exclude proline, which is a special case, and consider 20 different amino acids with an identical structure of the main chain (including selenomethionine). To describe all possible positions of the residue (N-terminal or C-terminal or in the middle of the chain) and major conformations (*cis* and *trans*) six different versions of each amino acid would be required,
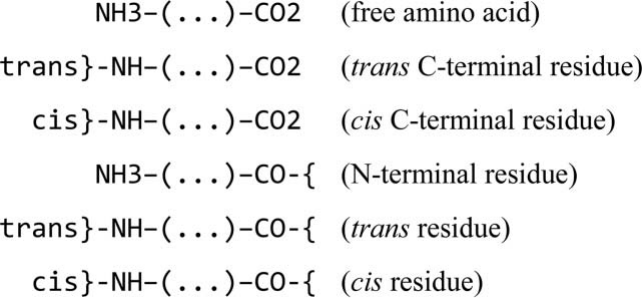


and this would amount to a total of 120 data blocks in the library. Such an approach does not require any additional type of data because the restraints associated with the covalent linkage between two amino acids are added to the description of one of them.

In the second approach, which is adopted in the restraint library, each amino acid is defined only once,^1^ 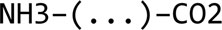


while the covalent linkages between amino acids,
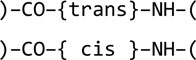


are defined separately using four library entries: two modifications
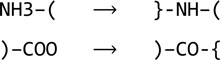


and two links,^2^ 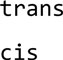


Such an approach requires two additional types of library entries. However, it substantially reduces the redundancy of the data stored in the library and, in our example, requires only 24 data entries instead of 120 for the description of all possible polypeptides containing the 20 amino acids.

Both links in the above example are generic from either side, *i.e.* neither the first nor the second monomer related by the link has to be a specific monomer, it only has to belong to a certain group (the group ‘peptide’ in this example). There are similar generic links for DNA and RNA polymers and also generic links for sugars that will be discussed in more detail later. Along with the generic links, there are also links that are generic from only one side. These include links associated with the peptide bonds between proline and other amino acids (PTRANS, NMTRANS, PCIS, NMCIS) and some of the terminal modifications of amino acids (*e.g.* NME_N-C for amidation of the C-terminus and FOR_C-N and ACE_C-N for formylation and acetylation of the N-terminus).^3^ This group also includes links for members of the group ‘pyranose’ with specific amino acids: Asn, Ser or Thr (NAG-ASN, NAG-SER and NAG-THR, respectively). There are also specialized links which can only be applied to two particular monomers, including one describing S—S bridges between cysteines.

Overall, the restraint library contains link and modification data for (i) polypeptide chains, (ii) S—S bridges, (iii) polynucleotide chains and (iv) glycosylated proteins. In addition, the restraint library contains modifications which are applied on their own and not in a context of any link for (i) some of the terminal peptides and nucleotides, (ii) methylated nucleotides and (iii) deprotonated states of phosphate and carboxyl groups. More details on the monomer library can be found on the CCP4 website in the *LibCheck* documentation.^4^

### 1.3. Generic links for sugars

The modification and link mechanism is even more important for the description of covalent linkages between sugar monomers. The number of possible combinations of these monomers is larger than that for amino acids. Each sugar monomer has more than two atoms through which links to other sugar molecules can be made. For example, in v.5.28 of the library there is a group of compounds called ‘pyranose’ (207 members) and any monomer of the group can undergo modification DEL-O1 (deletion of the O1 and HO1 atoms). Another sugar moiety can be modified by one of the following, DEL-HO2, DEL-HO3, DEL-HO4 or DEL-HO6 (deletion of an H atom), to make a corresponding link ALPHA1-2, ALPHA1-3, ALPHA1-4, ALPHA1-6 or BETA1-2, BETA1-3, BETA1-4, BETA1-6 with the first modified monomer.

**Figure 1:**
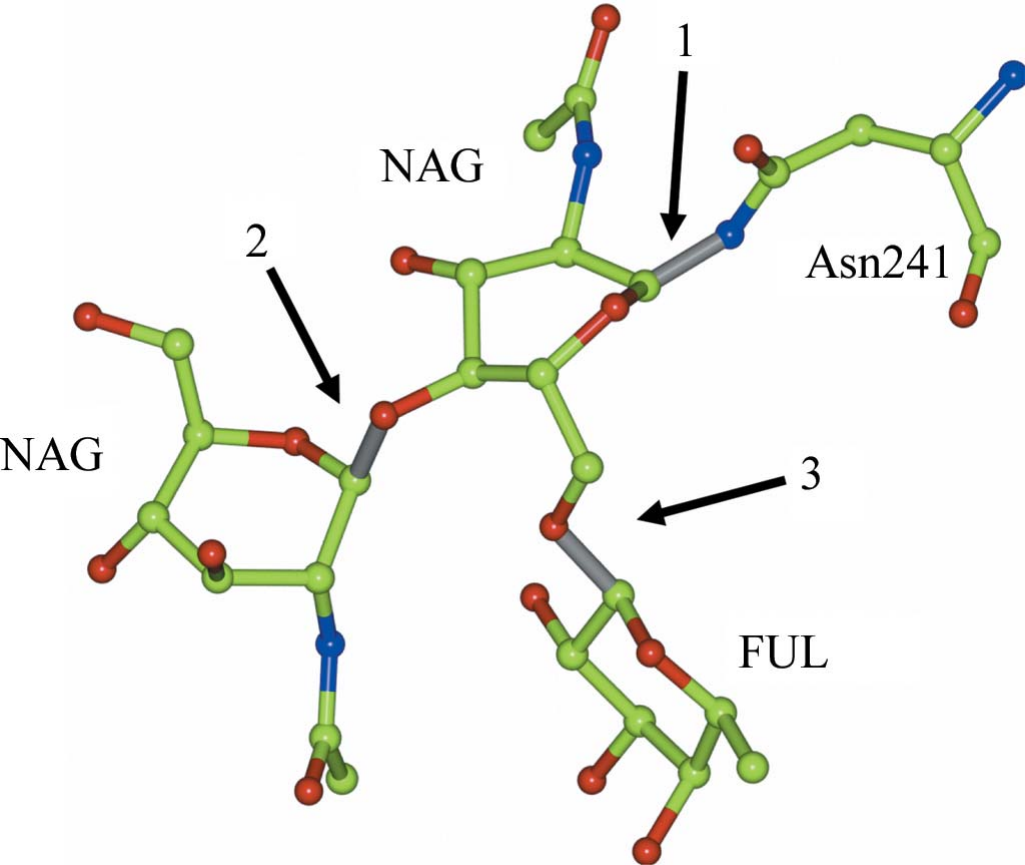
Example of glycosylation, which only requires the generic links for sugars defined in *REFMAC*’s standard monomer library for refinement. The figure shows one molecule of β-l-fucose (FUL) and two molecules of *N*-­acetyl-d-glucosamine (NAG) forming a polysaccharide covalently bound to Asn241 in the crystal structure of the G117H mutant of human butyrylcholinesterase (Nachon *et al.*, 2011 ▶; PDB entry 2xmb). Refinement of such a structure will implicitly use the links NAG-ASN (1), BETA1-4 (2), ALPHA1-6 (3) and associated modification from the restraint library. This figure and Fig. 3 ▶ were generated using *CCP*4*mg* (Potterton *et al.*, 2004 ▶).

Thus, for typical cases of glycosylation all the necessary modifications and links are present in the restraint library and by default *REFMAC* uses these library descriptions with no need for any additional instructions. An example of such a case is presented in Fig. 1 ▶. This is a crystal structure of mutant butyrylcholinesterase (Nachon *et al.*, 2011 ▶; PDB entry 2xmb) containing a simple branching polysaccharide with three residues. At this point it has to be mentioned that the PDB header LINK record conventions differ between the PDB and *REFMAC*. That from the PDB header lacks any reference to the link library entry. Therefore, if a PDB file has been downloaded from the PDB with the purpose of validation or electron-density analysis and the structure is to be re-refined using *REFMAC*, it is highly likely that *REFMAC* would assign the link type incorrectly, *e.g.* with incorrect target chiralities of the involved atoms. The easiest way to avoid this is removal of glycosylation-related LINK records from the file header (or removal of the whole header except for the CRYST1 record).

**Figure 2:**
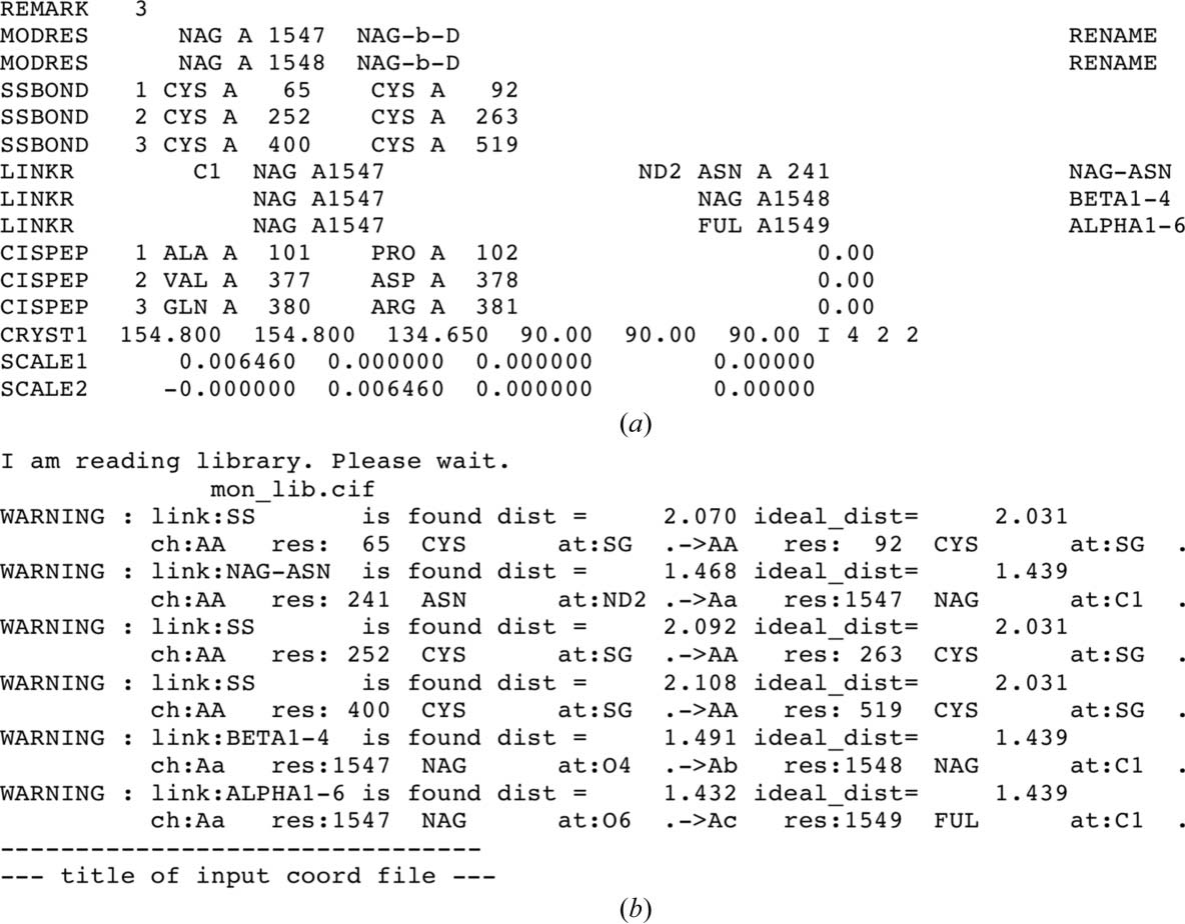
*REFMAC* checkpoints for refinement of glycosylated proteins. The figure shows fragments of (*a*) the output PDB file and (*b*) the *REFMAC* log file after successful refinement of the structure presented in Fig. 1 ▶. LINKR records indicate that *REFMAC* was able to associate short distances between monomers with library link entries and corresponding warnings in the log file prompt the user to check whether this assignment is correct and whether the linked atoms fit the electron density.

A more common scenario is building a polysaccharide into experimental electron density. It is always good practice to add sugar monomers one at a time and make sure that *REFMAC* has interpreted the user’s intention correctly. One possible problem could be the absence of the required link descriptions in the restraint library when the polysaccharide to be built is somehow special. In such a case the missing link can be defined by the user as described in §2. The main checkpoints are as follows: (i) *REFMAC* terminates normally, without error messages; (ii) the header of the output PDB file contains all necessary link records in *REFMAC* format; and (iii) the log file contains warnings saying that, for example, link ‘ALPHA1-6’ is found (Fig. 2 ▶). Such warnings prompt the user to check whether the correct types of links have automatically been assigned. Indeed, if sugar monomer(s) have not been very accurately positioned in the electron density then it is quite likely that *REFMAC* will assign an incorrect type of link between the new sugar monomer and its neighbour. In this case *REFMAC* will terminate normally. It will however write the incorrect type of link into the output PDB-file header and therefore the mistake will persist during further model building and refinement. In such a case the following checkpoints apply. Assignment of a correct link type means that (iv) the log file contains no warnings about significant deviation from the target chirality of sugar links and (v) no significant peaks are observed in the *F*_o_ − *F*_c_-type difference map near the linked atoms. If things do go wrong then changing the link type in the PDB-file header may help (*e.g.* from ALPHA1-3 to BETA1-3). Otherwise, more careful fitting of monomers into the electron density will be required or perhaps the choice of different sugar monomers.

## 2. User’s additional library of restraints

The (standard) restraint library is based on the PDB Chemical Components Dictionary that only contains the ligands found in deposited macromolecular structures and normally users do not have permissions to make modifications to it. Any new chemical features are to be stored in an additional user’s library. The two libraries are similar in that both may contain monomer, modification and link entries. They can be used in refinement simultaneously. In this case, the entries from the additional library take precedence over entries that have the same name in the standard library. This behaviour makes it possible, for example, to use updated versions of ligands.

The objective of *JLigand* is to generate or update the additional library.

### 2.1. Generation of template restraints for isolated ligands using *JLigand*

#### 2.1.1. Minimal and complete ligand descriptions

The restraint library may contain two types of ligand descriptions: minimal and complete (Vagin *et al.*, 2004 ▶). Along with the molecular graph, which incorporates lists of atoms and covalent bonds and their types, the minimal description contains types of chiral centres and atom names. In addition to this information, the complete description contains the target values and uncertainties for template restraints (covalent bond lengths and angles) and a list of planar groups. The *CCP*4 program *LibCheck* can extend the minimal ligand description to a complete description using the definitions of atom types, which are also included in the restraint library. Ligand descriptions (currently only complete descriptions) are stored in the restraint library as files in CIF format (Hall *et al.*, 1991 ▶). The minimal description and eventually the complete description can be derived from other molecular formats. In particular, SDF (Dalby *et al.*, 1992 ▶), *SYBYL* mol2 (Tripos, 2005 ▶) and PDB files, as well as SMILE strings (Weininger, 1988 ▶; Greaves *et al.*, 1999 ▶), can be used as input files for *LibCheck*. In addition to the complete ligand description, *LibCheck* outputs the Cartesian coordinates of the atoms that satisfy geometrical restraints from the complete description.

#### 2.1.2. Creating a complete ligand description

*JLigand* is a graphical interface for *LibCheck* (or potentially another similar application) that allows the visualization and editing of the minimal description and its transfer to *LibCheck* for creation of the complete description. Currently, *JLigand* can only be launched from the command line, but starting from *CCP*4 release v.6.3.0 it will be possible to launch it from the *CCP*4*i* interface. The set of editing operations that *JLigand* provides is typical of molecular editors, *i.e.* adding a covalently bound atom or a group, adding or deleting a bond or changing the bond type, deleting the atom and all of its bonds and changing the atom type, name or, if applicable, chirality type. Atomic coordinates are used to visualize the molecular graph. *Z* coordinates of the atoms and groups to be added are chosen in such a way that their addition takes place in the plane that is parallel to the screen and goes through the atom to which a new atom or group binds.

#### 2.1.3. Regularization procedure in *JLigand*

In *JLigand* the procedure of creating a complete ligand description is part of a ‘regularization’ procedure, which also includes the calculation of the atomic coordinates that satisfy template restraints from the complete description and are used in an updated three-dimensional presentation of the ligand. Apart from having a purely illustrative role, these coordinates can be saved as a PDB file and then used, for example, in *Coot* to model this ligand into the electron density. The complete description of the regularized ligand can be saved into a new additional library file or appended to an existing one.

A similar regularization procedure is carried out, albeit implicitly, when ligands are loaded from the restraint library or from external sources containing the molecular graph (SDF or SMILE). By default, atomic coordinates and restraints, if present in the input file, are ignored and only the molecular graph, atom names and chiralities, if specified, are used. The reason for such an approach is explained below (§2.3) and relates to generation of link library entries.

The regularization procedure includes two steps. Firstly, the information from the displayed ligand or from an input file is saved as a CIF file with minimal description and loaded into *LibCheck*, which generates a CIF file with complete description and a PDB file with atomic coordinates. Secondly, these coordinates are refined using *REFMAC* in idealization mode, with a CIF file being used as an additional library defining target values of geometrical restraints. The refined coordinates are then used to update or generate a graphical representation of the ligand that is being regularized or imported.

#### 2.1.4. Using coordinates for generation of ligand description

Another important option in *LibCheck* is the generation of a complete ligand description from atomic coordinates (a PDB file). In this case the minimal description is derived from coordinates and the molecular graph may be incorrect for coordinates with large uncertainty, although information on atom types contained in the PDB file helps in assignment of covalent bonds and their orders. With this option, chiralities of atoms are also derived from the coordinates and once the molecular graph has been generated and chiralities have been assigned the input coordinates are discarded. The subsequent procedure follows the same path as the generation of a complete description from minimal description.

There is yet another option of utilizing the input coordinates available in *LibCheck*. By default, target values for template restraints are derived from the restraint-library entry describing atom energy types, but these values can also be derived directly from the input coordinates. This option can be used for high-precision coordinates obtained by X-ray crystallography of small molecules, calculated using precise force fields or built using fragment libraries as in *CORINA* (Sadowski *et al.*, 1994 ▶) or *PRODRG* (Schüttelkopf & van Aalten, 2004 ▶). Such an option is also available in *JLigand*, but is not activated by default because it may result in modifications with too many edits (§2.2). To activate this possibility, one needs to start *JLigand* from the command line with the key ‘-as_is’. The menu element ‘File > Open As Is’ is then activated that contains submenus for SDF, PDB and CIF formats. CIF format is included because it corresponds to an additional library file which may also contain coordinates.

### 2.2. Covalent linkages made by amino acids

#### 2.2.1. Modified amino acids

Side chains of amino acids can make covalent linkages with polysaccharides, nonpolymer ligands, small groups or single atoms. Polysaccharides are diverse branching polymers and restraints for covalent linkages between their sugar monomers are most efficiently described by a link and modification mechanism (§1.3). A completely different mechanism is used to define restraints for linkages between amino acids and nonpolymers or single atoms. The whole compound including amino-acid and ligand moieties is described in the library as a single monomer. This monomer belongs to the group ‘peptide’ to ensure that it will be automatically incorporated into a polypeptide chain during refinement using generic links and modifications. There are 433 such special amino acids in the restraint library (the library was last synchronized with the PDB Chemical Components Dictionary at the end of 2010) and Table 1 ▶ presents the special amino acids most often encountered in the PDB. As can be seen, 80% of the special amino acids in Table 1 ▶ can be obtained by the binding of a small group(s) with one non-H atom to side-chain atom(s) of a ‘standard’ amino acid. Describing the addition of one or a few atoms using link library entries is not practical, but one can still describe differences with a standard amino acid using a single modification entry which is not associated with any link entry. However, in many cases special amino acids contain two distinct covalently bound moieties with an existing library description, one of which is a ‘standard’ amino acid. These special amino acids can alternatively be described using one link and two modification entries. There is one such example in Table 1 ▶, LLP, which can be represented as a link between Lys and PLP (see also §2.3).

The advantage of the last approach is that the amino-acid, ligand and atom names do not change, which makes more biological sense. However, this advantage is at the price of the changes in the linked compounds being assumed to be local­ized around the covalent linkage between the monomers. Actually, both approaches are sufficiently accurate for correct interpretation of electron density in most X-ray experiments, including those at high resolution.

#### 2.2.2. Covalent links between amino acids

As described previously, six links (four of which involve proline) and corresponding modifications represent all possible variations of the peptide bond, a dominant covalent interaction between amino acids in proteins. However, there are many infrequently occurring covalent linkages between side chains or between the side and main chains of two amino acids. These linkages are quite diverse and corresponding template restraints are not included in the restraint library. Importantly, description of such a linkage as a special amino acid is not possible, as such an amino acid would participate in not two but four peptide bonds.

**Figure 3:**
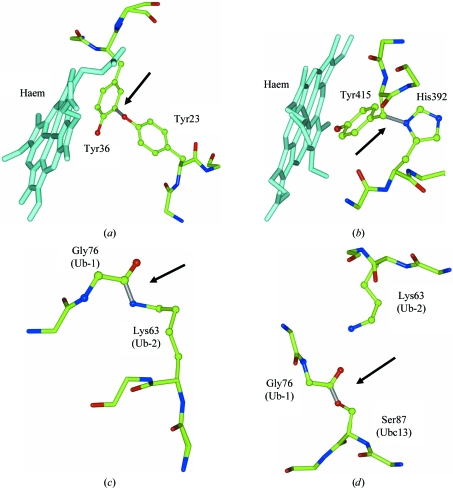
Examples of side chain to side chain and side chain to main chain covalent linkages in proteins (see citations and PDB codes in the main text): (*a*) Tyr–Tyr covalent link in *M. tuberculosis* haemoglobin O. (*b*) Tyr–His covalent link in HPII from *E. coli*. In both (*a*) and (*b*) the new covalent bond increases the rigidity of the haem site. (*c*) Lys to main chain (C-terminal Gly) link between ubiquitin molecules in the complex of the TAB2 protein with diubiquitin. TAB2 only binds linked ubiquitin molecules; the interaction is not shown, being distant from the link. (*d*) Covalent link between ubiquitin main chain and the side chain of Ser from the ubiquitin-conjugation enzyme Ubc13. Lys63 of the second ubiquitin is poised to attack the intermediate ester bond. Residues involved in covalent-linkage formation are shown in ball-and-stick representation. The linkages are shown in grey and are indicated by arrows.

The covalent linkages making the backbone of polypeptide or polynucleotide chains are not shown explicitly in the PDB coordinate files, while all others are shown in LINK records. The LINK records in the PDB entries available on 17 July 2011 were parsed to extract all special linkages between amino acids. (LINK records should not refer to C and N atoms of two amino acids or two cysteine S atoms and therefore all such cases were considered to be mistakes and were excluded from the analysis.) This search resulted in a total of 1279 examples distributed among 766 PDB entries and belonging to 571 different types of linkage. There were 365 unique examples, some of which might be artefacts, and 161 examples which occur several times in a single PDB entry. The most frequent nonpeptide linkages of Tyr with side and main chains of other amino acids are listed in Table 2 ▶. Two of these examples are shown in Fig. 3 ▶. In both cases the electron-density maps are well defined for the side chains involved in the covalent interactions. The link CE(TYR)–OH(TYR) is unique so far and can only be found in PDB entry 1ngk (Milani *et al.*, 2003 ▶) for *Mycobacterium tuberculosis* haemoglobin O (Fig. 3 ▶ *a*). It was suggested (Milani *et al.*, 2003 ▶) that this covalent link could form in the presence of oxidative species and might have a functional role in increasing the rigidity of the haem distal site. Such a modified residue, named di-isodityrosine, had been reported previously and proposed to promote insolubilization of plant cell-wall extensins (Brady *et al.*, 1996 ▶). Another example is the linkage CB(TYR)–ND1(HIS), with Tyr being involved in an interaction with Fe atoms coordinated by a haem. Such a link occurs in several PDB entries (Table 2 ▶), but all these are actually different crystal structures of the same protein, catalase HPII from *Escherichia coli*. One of these structures with PDB code 3p9p (Jha *et al.*, 2011 ▶) is shown in Fig. 3 ▶(*b*). The linkage was first identified by Bravo *et al.* (1997 ▶), who suggested that this covalent bond contributes to stability of the haem site. Similar to the previous case, the formation of this unusual modification was attributed to the attack of redox species.

A typical example of a side-chain to main-chain covalent linkage is that made by the C-terminal residue of ubiquitin (Gly76) to the side chain of Lys. There are at least 36 crystal structures in the PDB in which such linkages occur (and are listed in a LINK record). The maximal resolution obtained for the structure of a ubiquitinated protein was 1.18 Å in the crystal structure of the protein TAB2 in complex with di­ubiquitin (Sato *et al.*, 2009 ▶; PDB entry 3a9j; Fig. 3 ▶ *c*). TAB2 (transforming growth factor β-activated kinase 1 binding protein 2) specifically recognizes Lys63-linked polyubiquitin chains and this recognition is involved in critical cell-signalling pathways.

Even with ubiquitins there can be variations in the target amino acid, with a unique example of a linkage between ubiquitin and the OG atom of Ser from ubiquitin-conjugating enzyme (Ubc13) being reported by Eddins *et al.* (2006 ▶) (PDB entry 2gmi; Fig. 3 ▶ *d*). In this work, a C87S mutant was designed to capture an ester intermediate in which Gly96 from the ubiquitin C-terminus was covalently linked to Ser87 of Ubc13. This mimicked the formation of an intermediate Cys87(Ubc)–Gly96(first, donor ubiquitin) thioester bond before it is attacked by Lys63 of the next, acceptor ubiquitin and an isopeptide bond is formed as a step in the process of ubiquitin chain elongation. While this is a somewhat artificial situation, engineered bonds such as this are to be expected in many biological studies and this example highlights the importance of an additional library with case-specific template restraints.

### 2.3. Generation of template restraints for a linked monomer using *JLigand*

#### 2.3.1. Generation of link description

**Figure 4:**
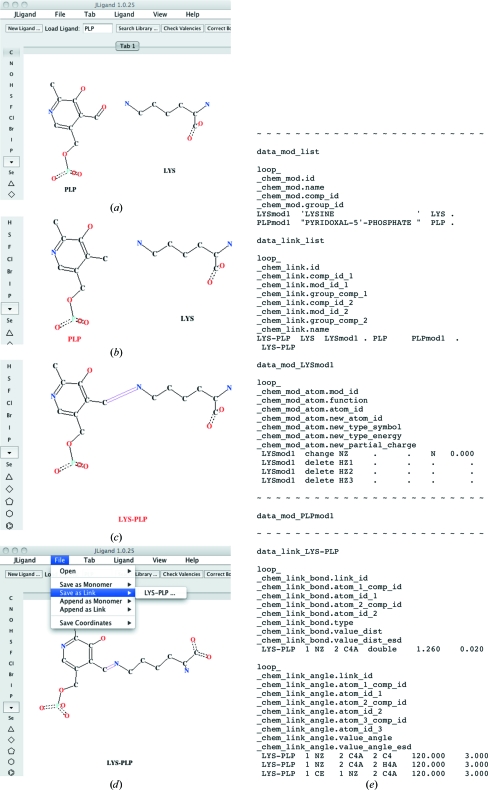
Defining restraints for the covalent linkage Lys–PLP using *JLigand*. Snapshots show the state of the *JLigand* interface after the following user actions: (*a*) loading monomers LYS and PLP from the restraint library, (*b*) removing O4A from PLP, (*c*) connecting C4A (PLP) with NZ (LYS) and defining the bond order and (*d*) regularization. The Save As Link menu has to be used to generate (*e*) an additional library file containing the following five data blocks: list of modifications, list of links, modifications LYSmod1 and PLPmod1 to be applied to LYS and PLP, respectively, and link LYS–PLP. The file header, tables of restraints for LYSmod1 and all the tables for PLPmod1 are omitted, as indicated by lines filled by tildes. H atoms are dealt with automatically. It is also possible to visualize them and handle them explicitly.

For a long time, new modifications and links were created using a text editor. The need for a convenient graphical tool for this work was the main incentive for the development of *JLigand*. The procedure of creating an amino acid–ligand link is presented in Fig. 4 ▶ using the example of a Schiff-base linkage between a lysine residue and a pyridoxal phosphate (PLP) resulting in the formation of an internal aldimine in aminotransferases (see, for example, Rhee *et al.*, 1997 ▶).

For a user, the procedure resembles conducting a chemical reaction *in silico*. Descriptions of both LYS and PLP are present in the restraint library. To make structure refinement possible, it is necessary to create an additional library with template restraints for the covalent linkage between LYS and PLP. The user types the three-letter codes of the compounds or performs a keyword search to load the compounds into *JLigand* (Fig. 4 ▶ *a*). In the next step, the user has to delete or add non-H atoms. In this example, the O4A atom of PLP has to be deleted as required for formation of the aldimine bond (Fig. 4 ▶ *b*). Finally, the covalent linkage and its bond order are defined (Fig. 4 ▶ *c*). After regularization, the new link can be saved in a new user’s additional library (File > Save As Link; Fig. 4 ▶ *d*) or appended to an existing additional library (File > Append As Link). Fig. 4 ▶(*d*) shows a graphical representation of the final result and Fig. 4 ▶(*e*) presents the content of the additional library listing required modifications to LYS and PLP monomers and restraints associated with the formation of a covalent linkage between the modified monomers.

An additional library thus obtained can be used for refinement of the structure where PLP is covalently bound to lysine. However, the generation of template restraints is only part of the overall procedure of crystal structure refinement including new ligands or links. The complete cycle typically includes the following steps: (i) the structure without ligands is refined, (ii) descriptions of ligands or links are generated, (iii) the ligands are positioned in the difference electron-density map and (iv) final refinement is performed for the model with the ligand using an additional library with required template restraints. The procedure is presented in detail in the *JLigand* tutorial, which can be found in the collection of tutorials in the Documentation section of the CCP4 web site.

#### 2.3.2. Creating a link: conventions and potential pitfalls

In the Lys–PLP example the standard library already contained the monomer descriptions. However, either one of the monomers or both of them can be created by the user and then joined with a covalent linkage during the same editing session. In such a case, it is assumed that the final version of the monomer description is that obtained from the last successful regularization. All changes made to the molecular graph after the final regularization and prior to joining the monomers with a link will be written into the additional library as a modification. A subsequent regularization of the linked monomers is usually accompanied by deletion or addition of H atoms. All such automatic changes are also added to the monomer modifications.

When the link is saved (File > Save As Link) all relevant data are stored: the descriptions of the link and two modifications and, if one or both monomers had been edited, the descriptions of these monomers as well. With such an approach many operations are carried out implicitly and do not distract the user’s attention with technical details of library organization. However, the user still has to be careful. To illustrate this point: in the example discussed above regularization of PLP after deleting O4A would have led to a new monomer description being written into the additional library under the same name (PLP). This would have blocked the PLP description from the standard restraint library, rendering the use of this additional library for the refinement of a structure containing both the internal aldimine and free PLP impossible (as, for instance, in PDB entry 2x5d; Oke *et al.*, 2010 ▶).

Such problems are inevitable when many operations are performed implicitly. Therefore, future versions of *JLigand* may provide a more transparent, although more sophisticated, interface explicitly showing lists of monomers, links and modifications present in the standard and user’s libraries.

#### 2.3.3. Algorithm for generating links and modifications

As in the case of monomers, the generation of template restraints and atomic coordinates for linked ligands is reduced to running *LibCheck* and *REFMAC*. The first step is the generation of the minimal description, which includes all the atoms and bonds inherited from modified monomers plus the bond that makes a covalent linkage. In the internal representation all atoms are identified by pointers on the objects associated with them, but representation in a CIF file is based on atom names. Therefore, in the regularization step atoms in the composite compound including two monomers are given unique names and the renamed compound goes through the *LibCheck*–*REFMAC* procedure in the same way as is performed for individual monomers (§2.1). The original atom names are then restored in the regularized composite compound. If a new H atom is added by *LibCheck*, the name for this atom is assigned based on the original name of a non-H atom to which it is connected. The graphical representation of the new composite compound is the same as that for the monomers. However, editing functions are blocked (the exception is the bond order of the covalent linkage between the monomers). This is aimed at avoiding over-complicated modifications.

On request (‘View’ or ‘Save’), but not before regularization, the description of the total compound is split into two modifications and one link. Initially, the template restraints involving atoms from different monomers are written into the link description and deleted from the description of the complete molecule, which therefore splits into two modified monomers. During editing, a reference to the parent atom from the initial ligand is remembered for each atom of the edited ligand; such a reference is missing for an atom added during editing. Therefore, the lists of deleted and added atoms and restraints can easily be restored after editing is finished. Besides, the atoms and restraints which have not been added or removed could have nevertheless changed. This is because the user might have changed the type of the atom or bond, or the atom and bond parameters might have changed during regularization because neighbouring atoms were added, deleted or modified. In the current version of *JLigand* the changes in atoms and bonds are traced by direct comparison of their old and new parameters. If an alteration in the type of atom or restraint has occurred or the changes in the target values of restraints have exceeded the standard deviation declared in the dictionary, the corresponding item is considered to be modified and therefore is added to the modification data block. Finally, the decision is made on the original monomers to which modifications were applied. If these are available from the main or additional restraint library their descriptions are discarded. Otherwise, one or both monomers are added to the additional library.

#### 2.3.4. Drawbacks of the existing algorithm

If the descriptions of the original monomers were generated by another program or against a different set of atom types or derived from atomic coordinates, then such an algorithm would result in too many edits in the modification entries. This is why by default all the imported monomer data are reduced to a minimal description and the complete description is then generated from scratch in the same manner as is performed during the implicit generation of the description representing linked monomers. This in particular is the reason why the option ‘Read As Is’ is hidden by default and alternative regularization protocols (using, for example, *PRODRG* for generation of restraint parameters) have not yet been implemented. It is anticipated that in future versions of *JLigand* the criterion of bond ‘alteration’ would be changed. The decision on inclusion of the atom or restraint in the modification will be based on changes in the molecular graph, *e.g.* defined by whether any changes occurred in the first and second co­ordination spheres of the atom or restraint in question.

## 3. Conclusions

*JLigand* is a new *CCP*4 program for three-dimensional editing of molecular graphs, the generation of corresponding template restraints and their storage in the additional library file, which can be used for model building and refinement together with the standard restraint library. The molecular graph is generated from scratch or from the atomic coordinates (PDB file) or imported from external sources such as a SMILE string, CIF library file or SDF file and edited if necessary. The generation of restraints from a molecular graph is performed using *LibCheck*. Atomic coordinates are regularized using *REFMAC* and are used to display the new molecule.

Two other types of entries that are present in the restraint library and may also be present in the additional library are modifications and links. 32 modifications from the standard library are used for the generation of restraints for modified monomers, while another 21 modifications and 65 links describe changes and additional template restraints that are necessary to assemble appropriate monomers into polypeptides, polynucleotides or polysaccharides. This small number of standard library entries accounts for all or almost all of the covalent linkages between monomers in a given structure, while there could be a few novel linkages that are specific to a limited number of structures. Even quite a superficial analysis of the PDB showed that these specific linkages could be highly diverse. Providing the graphical and computational tools required for the generation of such descriptions is a distinctive feature of *JLigand*.

The Java executable, source code and tutorials for *JLigand* 5 will be available from CCP4 starting with v.6.3.0.

## Figures and Tables

**Table 1:** Special amino acids most frequently used in the PDB as of July 2011 with their names in the restraint library Frequency of occurrence was calculated by counting LINK records in the PDB file header.

Three-letter code	Compound name	No. of PDB files	Total in all PDB files
SEP	Phosphoserine, *O*-phospho-L-serine	381	757
TPO	Phosphothreonine, *O*-phospho-L-threonine	326	590
PTR	Phosphotyrosine, *O*-phospho-L-tyrosine	270	517
CSO†	Cysteine sulfenic acid, *S*-oxycysteine	254	531
KCX	Lysine NZ-carboxylic acid, NZ-formyllysine	229	573
LLP	*N*′-Pyridoxyl-lysine-5′-monophosphate	221	448
CSD‡	Cysteine sulfinic acid	167	330
CME	*S*,*S*-(2-Hydroxyethyl)thiocysteine	150	554
TYS	Tyrosine-*O*-sulfate, sulfonated tyrosine	140	161
DAL	D-Alanine	122	315
M3L	*N*-Trimethyllysine	118	165
MLY	*N*-Dimethyllysine	115	4272
OCS	Cysteine sulfonic acid	99	225
ABA	α-Aminobutyrate, α-aminobutyric acid	83	262
CSW‡	Cysteine sulfinic acid	73	147
ALY	NZ-Acetyllysine	70	106
CSX†	*S*-Oxycysteine, cysteine sulfenic acid	63	120
TPQ	2,4,5-Trihydrophenylalanine quinone	53	117
HIC	4-Methylhistidine	50	75
DVA	D-Valine	50	249

† The oxidized cysteine compound entries CSO and CSX (cysteine sulfenic acid) are equivalent apart from the restraint library’s interpretation of the type of S—O bond (single or double).

‡ The same applies to entries CSD and CSW for cysteine sulfinic acid.

**Table 2:** Occurrences of Tyr paired with any amino acid in LINK records in the PDB Pairs contacting by atoms with names C and N are excluded. The indicated pairs of atoms make close contacts in the range 1.14–2.22 Å in one or more PDB files. Fig. 3 ▶ presents two examples from this list, a relatively frequent one and a unique one, both corresponding to true covalent linkages. However, the full list has not been validated and may contain artefacts.

Atoms in PDB link records				No. of PDB files	Total in all PDB files
TYR	CE2	HIS	NE2	27	52
TYR	CE2	MET	SD	18	35
TYR	CB	HIS	ND1	14	56
TYR	CE1	TRP	CH2	14	27
TYR	CE2	CYS	SG	7	14
TYR	C	SER	OG	5	6
TYR	CE1	GLN	CG	3	6
TYR	CE1	CYS	SG	3	4
TYR	OH	TYR	CE2	1	6
TYR	CE1	HIS	NE2	1	4
TYR	CE1	SER	O	1	4
TYR	CZ	SER	C	1	4
TYR	CZ	SER	N	1	4
TYR	CE1	ASP	N	1	3
All 52 combinations				135	274

## Footnotes

1 In the restraint library, 21 ‘standard’ amino acids are described as being bound in the middle of a polypeptide chain, without OXT, HN1 and HN2 atoms but with HN atoms. This is reverted to a description of free unbound amino acids when the library is being read. All the ‘special’ amino acids (see §2.2) as well as nucleotides and sugars are presented by descriptions of their free forms. The ‘standard’ amino acids are kept in their polymerized form for compatibility reasons and can be reverted to their free forms straight away with no changes in the *REFMAC* code.

2 The modifications DEL-HN1 and DEL-OXT required for TRANS or CIS links are applied based on the position of the amino acid in the polypeptide chain, *i.e.* are applied if the chain contains residues with smaller or larger sequence number, respectively. Therefore, TRANS, CIS and several other links do not contain references to these modifications.

3 Some monomer entries represent groups rather than free compounds and do not require further modification for their incorporation into a polymer chain, such as, for example, monomer entry FOR representing the formyl group, which can terminate the polypeptide chain.

4 See also http://www.ysbl.york.ac.uk/~alexei/dictionary.html.

5
*JLigand* can currently be downloaded from the YSBL site at http://www.ysbl.york.ac.uk/mxstat/JLigand/.
